# A rare case report of giant epicardial lipoma compressing the right atrium with septal enhancement

**DOI:** 10.1186/s13019-015-0375-x

**Published:** 2015-11-05

**Authors:** Shengjun Wu, Peng Teng, Yuhan Zhou, Yiming Ni

**Affiliations:** Department of Cardiothoracic Surgery, the First Affiliated Hospital of Zhejiang University, 79#, Qingchun Road, Hangzhou, Zhejiang 310000 China

**Keywords:** Cardiac lipoma, Right atrium, Local mass effect, Tumor septa

## Abstract

**Background:**

Cardiac Lipoma is a rare entity constituting approximately 10-19 % of primary tumors of the heart and pericardium. To our best knowledge, such a large cardiac lipoma with septal enhancement in our case has never been reported before.

**Case presentation:**

Here we present a rare case of a 65-year-old symptomatic female with an unusual giant cardiac lipoma. Due to the enhancement of the tumor septa, it was first diagnosed as liposarcoma and thought to be unresectable. Debulking surgery was performed to release patient’s symptoms.

**Conclusions:**

The patient ultimately underwent complete tumor resection with uneventful postoperative evolution. The postoperative pathological diagnosis is cardiac lipoma.

## Background

Primary cardiac tumors are extremely rare with a reported incidence of 0.17-0.19 % at autopsy. Cardiac lipoma constitutes approximately 10-19 % of primary tumors of the heart and pericardium [1]. Cardiac lipoma commonly originates from the epicardial adipose tissue of the right atrium (RA) and left ventricle (LV). We report an unusual case of a giant epicardial lipoma with septal enhancement which was first diagnosed as liposarcoma. As far as we know, giant cardiac lipoma with similar imaging findings has seldom been reported.

## Case presentation

A 65-year-old Chinese female, without any positive medical and family history, presented with recurrent chest discomfort on exertion for more than 10 years. Physical examination and laboratory tests were unremarkable.

**Fig 1:**
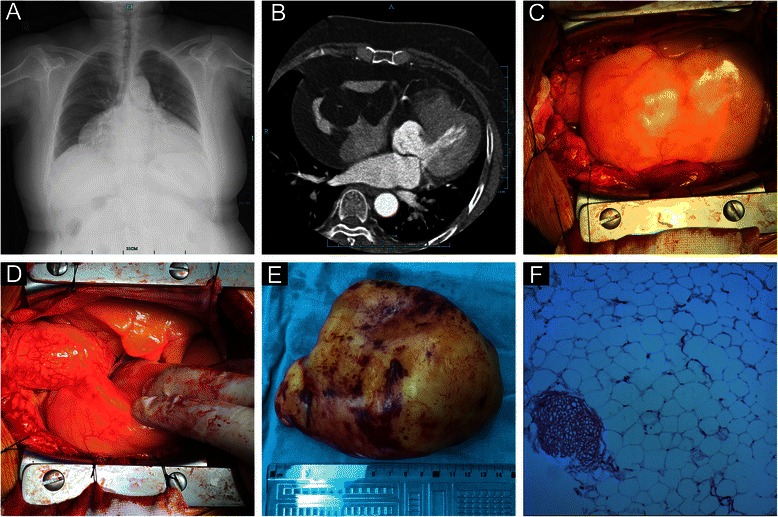
**a** Chest roentgenogram showed the water-bottle heart with enlarged cardiac silhouette. **b** Enhanced CT showed an intrapericardial low-density tumor encompassing the free wall of right atrium. The tumor was well circumscribed and the septa of the tumor presented with enhancement. **c**, **d** A giant lipoid mass was noted after pericardiotomy, which was yellow, soft and well-encapsulated. **e** The resected tumor measured about 14 × 11 cm, weighted about 450 g and was well-encapsulated. **f** Histopathological study confirmed the diagnosis of lipoma (H&E stain)

On admission, the water bottle sign of the cardiac silhouette was found on the chest roentgenogram (Fig. 1a). Transthoracic echocardiography (TTE) revealed a large (10.2 × 7.5 × 3.8 cm) well-defined homogenous hypoechoic mass located in the pericardial cavity, encompassing the anterior walls of aorta and pulmonary artery. Further assessment by enhanced computed tomography (CT) showed a large lobulated low-density mass in the pericardial cavity (Fig. 1b). The tumor presented with septal enhancement and absence of parenchymal enhancement. Meanwhile, computed tomographic angiography (CTA) of the heart confirmed normal coronary arteries and no compression signs by the tumor. After extensive workup, a preoperative diagnosis of cardiac liposarcoma was made based on the extensive growth and the enhancement of the tumor septa, which made us believe complete tumor resection was of high difficulty. Debulking surgery was scheduled to release patient’s symptoms.

Intraoperatively, a giant lipoid tumor originating from RA was noted after pericardiotomy (Fig. 1c). The tumor was based predominantly on the entire free wall of the RA, stretching down from the RA appendage all the way to the orifice of the inferior vena cava (Fig. 1d). The RA was extremely deformed owing to the significant local mass effect. Due to well capsulation and no signs of local invasion, the tumor was considered a lipoma and therefore resectable. Complete tumor resection was achieved during beating heart surgery with cardiopulmonary bypass assistance. Excision required removal of most of the RA free wall. Direct suture for RA defect seemed to be impossible because of the large defect size and the high tension of suture. In addition, the deformation of RA could results in an unsatisfactory long-term outcome such as systemic congestion. The RA wall that remained was ultimately repaired with a 7 × 5 cm autologous pericardial patch. After declamping the superior and inferior vena cava, the RA pressure was back to normal according to right heart catheterization. Postoperative transesophageal echocardiography also confirmed normal right heart function.

The resected lipoma was about 14 × 11 cm and 450 g (Fig. 1e). The histopathological examination made the final diagnosis of cardiac lipoma (Fig. 1f). The patient had an uneventful recovery and without any recurrence at 1-year follow-up.

## Discussion

Cardiac lipoma was first described in 1856. It is extremely rare with a reported incidence of about 10-19 % among primary tumors of heart and pericardium [1]. Histopathologically, cardiac lipoma can be classified into two types: lipomatous hypertrophy of the interatrial septum and true lipoma. The former one is a nonencapsulated mass of adipose tissue which is usually in continuity with the epicardial fat [2]. The true lipoma is a neoplasm, constituted of encapsulated masses of adipose tissue, typically mature adipocytes [3].

Cardiac lipoma can occur throughout the heart, including pericardium, epicardium, endocardium and myocardium. The most common chambers involved are LV and RA. According to the literature, cardiac lipoma has no sex or age predominance and is extremely variable in size [1]. The lipoma in our case was approximately 14 cm which had not been reported before. Patients with cardiac lipoma are usually asymptomatic until local mass effect lead to hemodynamic changes and cardiac insufficiency. Symptoms commonly reported are fatigue, dyspnea, palpitation, syncope and even chest pain, which are frequently a result of coronary artery or cardiac conductive system involvement. Sudden death case caused by cardiac lipoma has also been reported [4].

Transthoracic echocardiography is a valuable initial investigation for detection and diagnosis of cardiac tumors, due to its relatively high accuracy and non-invasiveness. Enhanced CT and magnetic resonance imaging (MRI) are of utmost importance and value in providing information regarding size, location and nature of the tumor. Cardiac lipoma can be diagnosed with high certainty due to their typical imaging characteristics. Typically in MRI, lipoma has homogenous high signal intensity in both T1- and T2- weighted images when compared with myocardium. Homogeneous low density without enhancement after contrast infusion is another typical manifestation of lipoma in enhanced CT, which is different from liposarcoma. Commonly, liposarcoma can be excluded by enhanced CT because it possesses higher density and enhancement after contrast infusion. In our case, the tumor parenchyma presented with homogenous low density without enhancement while the tumor septa manifested with high density and enhancement. Its mixed properties of lipoma and liposarcoma made our case unique, which also explained the preoperative misdiagnosis. Preoperative evaluation of coronary artery by coronary angiography or CTA is necessary in order to exclude their affection by tumor.

There is consensus about surgical treatment in symptomatic patients with cardiac lipoma, but it is still controversial whether performing surgery in asymptomatic patients. Some scholars prefer clinical follow-up in patients in whom the lipoma is found incidentally, due to its very low growth rate. Surgical resection for cardiac lipoma is the optimal choice and generally offers good long-term outcomes [5].

## Conclusions

Cardiac lipoma is a rare entity which constitutes only a few percent of primary cardiac tumors. This case report describes a rare giant epicardial lipoma presenting with septal enhancement which has not been reported before. TTE, coronary angiography and especially enhanced CT and MRI are helpful for diagnosis. For symptomatic patients, surgical treatment is the optimal therapeutic option and it is associated with favorable long-term outcomes.

## Consent

Written informed consent was obtained from the patient for the publication of this report and any accompanying images.

## Abbreviations

CT: computed tomography
CTA: computed tomographic angiography
LV: left ventricle
MRI: magnetic resonance imaging
RA: right atrium
TTE: transthoracic echocardiography
